# Clinical Images in Emergency Medicine: Cushing’s Disease

**DOI:** 10.5811/cpcem.20780

**Published:** 2024-04-09

**Authors:** Jason D. Vadhan, Nathaniel Hansen, Fernando L. Benitez, Larissa I. Velez

**Affiliations:** UT Southwestern Medical Center, Department of Emergency Medicine, Dallas, Texas

**Keywords:** Cushing’s disease, Cushing syndrome, pituitary adenoma, case report

## Abstract

**Case Presentation:**

A 22-year-old female presented to the emergency department with a two-month history of worsening fatigue, unintentional weight gain, and progressive facial swelling. Physical examination findings included hirsutism, moon facies, and abdominal striae. Subsequent brain magnetic resonance imaging revealed the presence of a 2.4-centimeter pituitary macroadenoma, confirming the diagnosis of Cushing’s disease. The patient was then admitted for neurosurgical tumor resection.

**Discussion:**

Cushing’s disease is exceedingly rare and often presents with symptoms resembling more prevalent disorders, contributing to delays in diagnosis. Therefore, maintaining a high index of suspicion for this disease is crucial for emergency physicians.

CPC-EM Capsule What do we already know about this clinical entity?

*Cushing’s disease, caused by pituitary adenomas, often leads to delayed diagnosis due to nonspecific symptoms.*

What makes this presentation of disease reportable?

*This report highlights the classic clinical presentation of Cushing’s disease including distinctive physical exam findings.*

What is the major learning point?

*Maintaining a high index of suspicion for rare diseases like Cushing’s in the emergency department is essential for timely diagnosis.*

How might this improve emergency medicine practice?

*Prompt recognition of Cushing’s disease symptoms can lead to a timely diagnosis and appropriate definitive care, ultimately improving patient outcomes.*

## CASE PRESENTATION

**Image 1.**
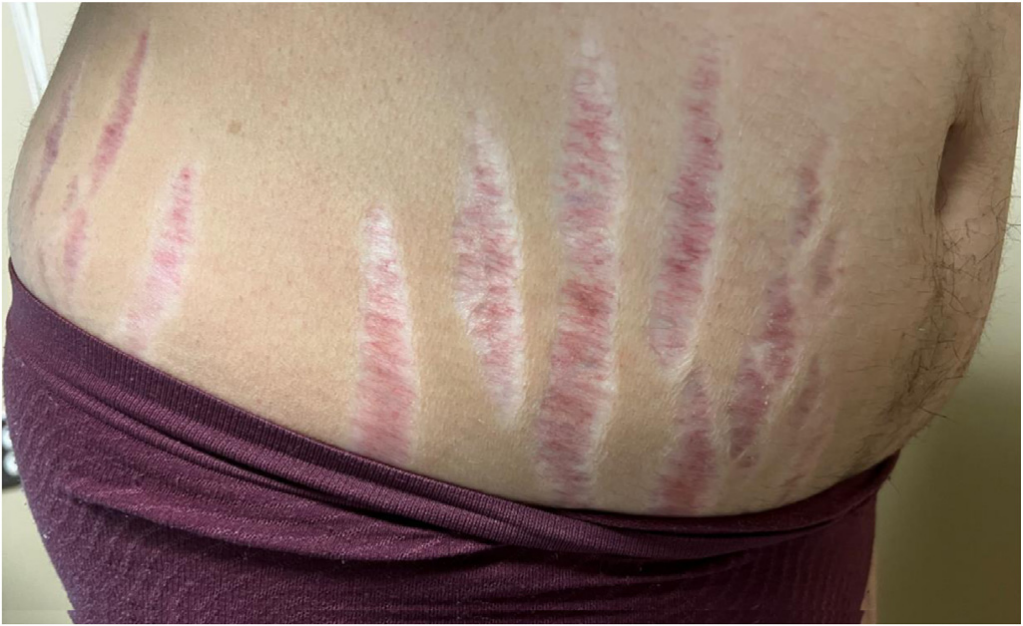
Lower abdominal striae due to hypercortisolism.

**Image 2.**
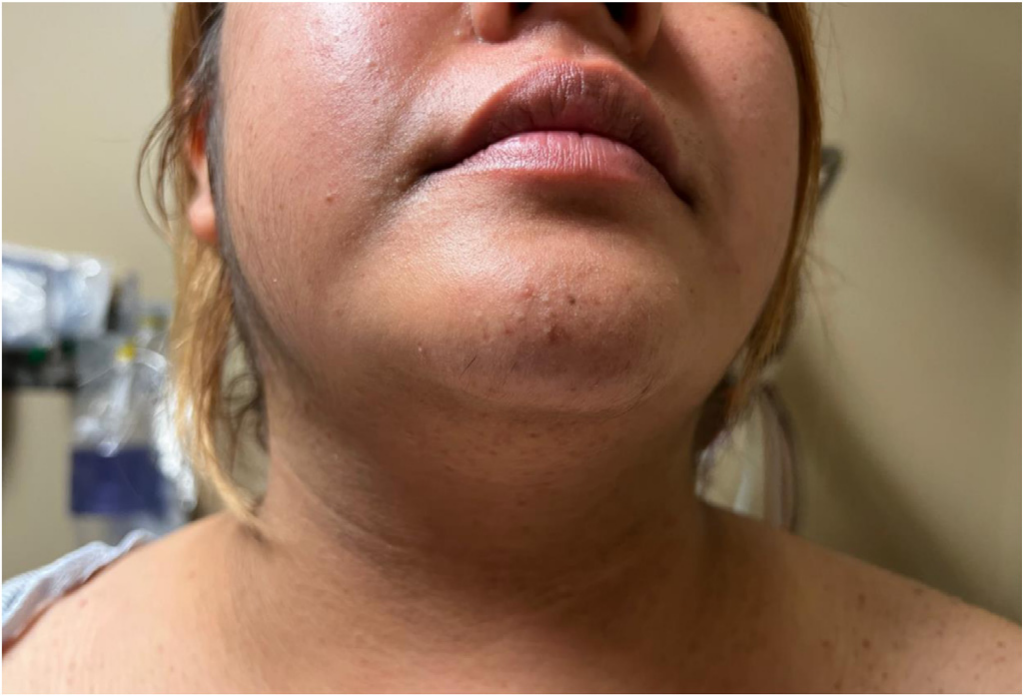
Facial and trapezius adiposity, colloquially referred to as “moon facies” and a “buffalo hump.”

**Image 3.**
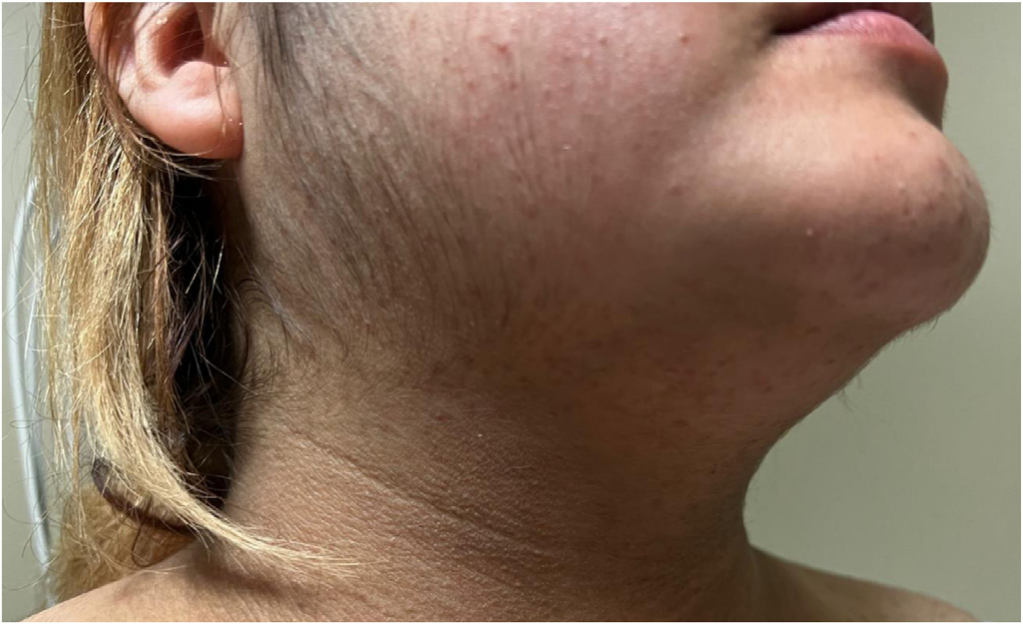
Hirsutism of the face due to increased adrenocorticotrophic hormone production, resulting in hyperandrogenism.

A 22-year-old female with a past medical history of hypertension and diabetes presented to the emergency department with two months of abdominal striae, persistent fatigue, unintentional weight gain exceeding 30 pounds, and progressive facial swelling. Physical exam revealed the presence of abdominal striae (Image 1), facial and trapezius adiposity (Image 2), and hirsutism (Image 3). Since the patient was not receiving steroid therapy at the time, her symptoms raised suspicion for Cushing’s disease. Subsequently, a brain magnetic resonance imaging (MRI) with intravenous contrast was performed, revealing a 2.4-centimeter pituitary macroadenoma causing severe upward displacement of the optic chiasm (Supplementary Image). Neurosurgery was sought, and the patient was admitted for operative management.

## DISCUSSION

Cushing’s disease is a rare disorder characterized by excessive cortisol production from the adrenal glands, which can either be from the adrenals directly or from corticotropin-releasing tumors in the lungs or pituitary gland.^1^ The term “Cushing’s disease” refers explicitly to the presentation of Cushing’s syndrome caused by a pituitary tumor. Cushing’s disease is more commonly observed among women, typically appearing between 20–40 years of age.^1^ Clinical manifestations are attributed to increased cortisol production, which causes weight gain, fatigue, poor concentration, hypertension, hyperglycemia, excess hair growth, abdominal striae, adipose deposition, and menstrual irregularity.^2^ Unfortunately, these symptoms are nonspecific and overlap with common medical conditions such as diabetes, hypertension, and polycystic ovarian syndrome. Consequently, the diagnosis of Cushing’s disease is often delayed, with an average time to diagnosis exceeding three years from symptom onset.^3^

The evaluation for Cushing’s disease is typically initiated in an outpatient setting and involves various tests, including midnight salivary cortisol measurement, low-dose dexamethasone suppression test, or 24-hour urine-free cortisol level assessment.^2^ However, in the emergency department, obtaining a brain MRI may be warranted to detect a visible pituitary tumor, which can be seen approximately 50% of the time, as in this case.^2^ When pituitary tumors are discovered, neurosurgical consultation and operative resection are often necessary.

## Supplementary Information


