# Supplementary material for: Clinical Images in Emergency Medicine: Cushing’s Disease
Source: Clin Pract Cases Emerg Med. 2024 Apr 9;8(2):174–5. doi: 10.5811/cpcem.20780 (PMC11166076; doi:10.5811/cpcem.20780)
Supplement: Supplementary file 1 [file cpcem-8-174-s001.docx]

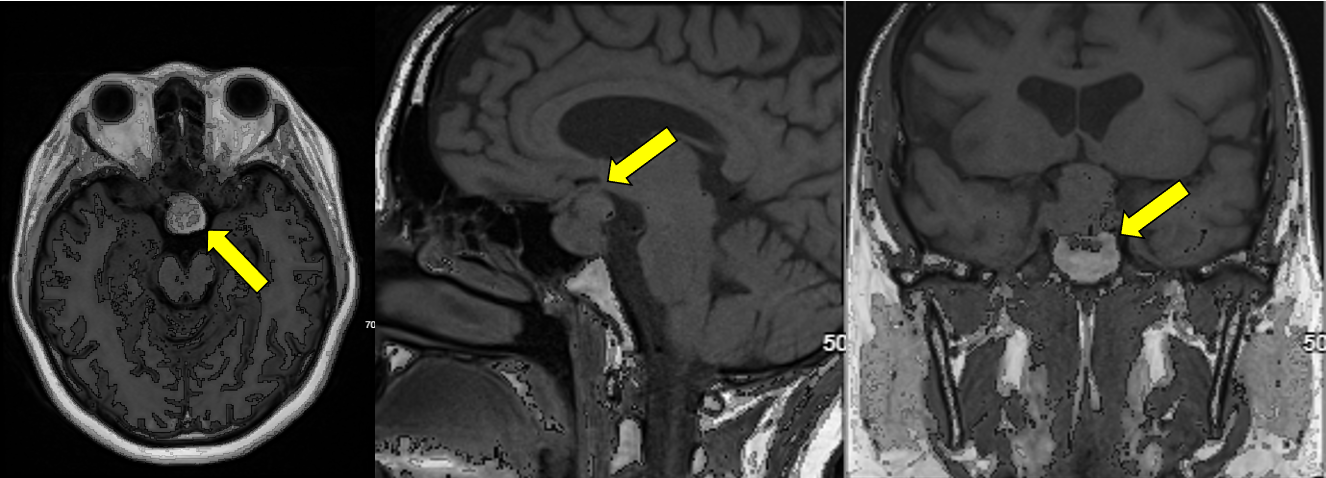


**Supplementary Image 1.** Axial, Sagittal, and Coronal T1 images demonstrating a 2.4 cm pituitary macroadenoma (arrows) with a severe upward displacement of the optic chiasm and associated microhemorrhage.
